# Crystal Structure of TDRD3 and Methyl-Arginine Binding Characterization of TDRD3, SMN and SPF30

**DOI:** 10.1371/journal.pone.0030375

**Published:** 2012-02-17

**Authors:** Ke Liu, Yahong Guo, Haiping Liu, Chuanbing Bian, Robert Lam, Yongsong Liu, Farrell Mackenzie, Luis Alejandro Rojas, Danny Reinberg, Mark T. Bedford, Rui-Ming Xu, Jinrong Min

**Affiliations:** 1 Hubei Key Laboratory of Genetic Regulation and Integrative Biology, College of Life Science, Huazhong Normal University, Wuhan, People's Republic of China; 2 Structural Genomics Consortium, University of Toronto, Toronto, Ontario, Canada; 3 National Laboratory of Biomacromolecules, Institute of Biophysics, Chinese Academy of Sciences, Beijing, People's Republic of China; 4 Howard Hughes Medical Institute, Department of Biochemistry, New York University School of Medicine, New York, New York, United States of America; 5 The University of Texas MD Anderson Cancer Center, Science Park-Research Division, Smithville, Texas, United States of America; 6 Department of Physiology, University of Toronto, Toronto, Ontario, Canada; University of Cambridge, United Kingdom

## Abstract

SMN (Survival motor neuron protein) was characterized as a dimethyl-arginine binding protein over ten years ago. TDRD3 (Tudor domain-containing protein 3) and SPF30 (Splicing factor 30 kDa) were found to bind to various methyl-arginine proteins including Sm proteins as well later on. Recently, TDRD3 was shown to be a transcriptional coactivator, and its transcriptional activity is dependent on its ability to bind arginine-methylated histone marks. In this study, we systematically characterized the binding specificity and affinity of the Tudor domains of these three proteins quantitatively. Our results show that TDRD3 preferentially recognizes asymmetrical dimethylated arginine mark, and SMN is a very promiscuous effector molecule, which recognizes different arginine containing sequence motifs and preferentially binds symmetrical dimethylated arginine. SPF30 is the weakest methyl-arginine binder, which only binds the GAR motif sequences in our library. In addition, we also reported high-resolution crystal structures of the Tudor domain of TDRD3 in complex with two small molecules, which occupy the aromatic cage of TDRD3.

## Introduction

Arginine methylation is an abundant covalent post-translational modification, which regulates diverse cellular processes, including transcriptional regulation, RNA processing, signal transduction and DNA repair [1]. There are three types of arginine methylation, i.e., monomethylarginine (Rme1, or MMA), asymmetric dimethylarginine (Rme2a, or aDMA) and symmetric dimethylarginine (Rme2s, or sDMA). To date, nine protein arginine methyltransferases (PRMT) have been identified in the human genome, and they can be grouped into three classes. Type I PRMTs (PRMT1, 2, 3, 4, 6, and 8) generate both monomethylarginine and asymmetric dimethylarginine modifications. Type II PRMTs (PRMT5 and 7) generate monomethylarginine and symmetric dimethylarginine modifications. The only known type III PRMT generating only monomethylarginine mark is PRMT7. Additionally, an atypical type IV PRMT methylates the internal guanidine nitrogen atom, which is only identified in yeast [1].

PRMTs can methylate a variety of target proteins, including histones, Sm proteins and transcription factors [1], [2], [3]. Many of these target proteins contain glycine and arginine-rich (GAR) motifs, such as SmD1/3 and MIWI/PIWIL proteins [4], [5]. Some target proteins harbor PGM motifs [6]. Arginine residues within the GAR and PGM motifs are the methyl-acceptor sites. Arginine methylation can both positively and negatively regulate protein-protein interactions of the target proteins. For examples, histone H3R2 methylation by PRMT6 prevented methylation of H3K4 by the MLL family of histone H3K4 methyltransferase complexes [7]. In addition, histone H3R2 methylation also blocks the binding of H3K4me effectors, such as WDR5 [8], [9] and BPTF [10] from recognizing the H3K4me3 mark [7], [11], [12]. On the other hand, arginine methylation can also create docking sites to foster protein-protein interaction. So far, the Tudor domain is the only known effector domain that is able to recognize methyl-arginine marks.

The Tudor domain is the founding member of the Tudor domain ‘Royal Family’, which includes chromodomain, MBT repeat domain and the PWWP domain [13]. Many members in this family have been shown to bind lysine-methylated histones and non-histone proteins [14], [15]. Some Tudor domains have also been shown to bind methylated lysine [16], [17]. However, Tudor domains are better known for binding methyl-arginine marks [4], [18], [19], [20], [21]. In 2001, Friesen *et al* showed that the SMN (survival of motor neurons) protein binds dimethylated GAR motifs of SmD1 and SmD3 via its Tudor domain [4], [22]. Another study shows that SMN also binds methylated PGM motifs within CA150, SAP49, SmB and U1C proteins, which are specifically methylated by CARM1 [6]. SMN is a protein essential for biogenesis of small nuclear ribonucleoproteins and its deficiency causes spinal muscular atrophy disorders. In 2005, Cote and Richard demonstrated that the Tudor domains of SMN and SPF30 (Splicing factor 30 kDa, or SMNDC1, Survival motor neuron domain-containing protein 1) and TDRD3 preferentially recognize symmetrical dimethylated arginine motifs in proteins, and arginine methylation and subsequent Tudor protein recruitment is potentially important for the proper assembly and localization of Sm proteins [18]. Through a protein domain microarray, Yang et al recently discovered that TDRD3 also functions as a arginine-methylated histone reader, which preferentially recognizes H3R17me2a and H4R3me2a marks [21]. Interestingly, these histone sequences do not contain either GAR or PGM motifs.

Although the Tudor domains of TDRD3, SMN and SPF30 have been demonstrated to be methyl-arginine binders for a number of years, their binding specificity and affinity has not been studied systematically and quantitatively, and the molecular mechanism for the recognition of methyl-arginine by their Tudor domains remains elusive. The only structurally characterized interactions between a Tudor domain and a methylated arginine involve recognition of symmetrically dimethylated arginines of PIWI/MIWI proteins [19], [20]. In this report, we systematically characterized the binding specificity and affinity of the Tudor domains of these three proteins quantitatively, and report high resolution crystal structures of the Tudor domain of TDRD3 with two small molecules, which provides important insights into the structural basis of the methyl-arginine recognition by the Tudor domain.

## Results and Discussion

### TDRD3 preferentially recognizes asymmetrical dimethylated arginine mark

**Figure 1:**
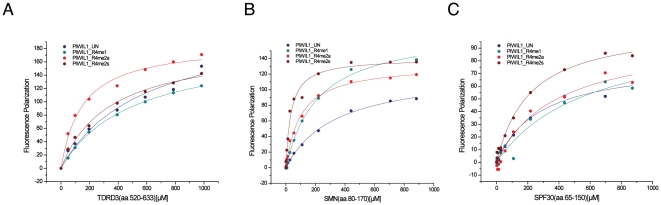
Fluorescence polarization binding curves of TDRD3 (aa 520–633), SMN (aa 80–170), and SPF30 (aa 65–150) to the PIWIL1-R4 peptides. The buffer used in the fluorescence polarization assay is 20 mM Tris pH 7.5, 50 mM NaCl, 1 mM DTT and 0.01% Triton X-100. The data are measured at 25°C and corrected for background by subtracting the free-labeled peptide background. The Kd values are the average of three independent measurements.

TDRD3 contains a Tudor domain at its C-terminus. The Tudor domain of TDRD3 has been shown to recognize arginine-methylated histones and Sm proteins [18], [21]. In order to characterize its binding specificity and affinity quantitatively, we performed a series of fluorescence polarization (FP) binding assays using our fluorescein-labeled peptide library, which includes GAR motif-containing SmD3 and PIWIL1 peptides, PGM motif-containing SmB peptides, and histone H3R2 peptides (Table 1 and Fig. 1). A low salt concentration (50 mM NaCl) is used in the FP binding assay due to the weak binding affinities of these proteins to their ligands. At this salt concentration, the binding affinities are increased about four times compared to the data measured at 200 mM NaCl (Fig. S1), which will save the reagents and make the Kd measurement more reliable. Our data show that TDRD3 (residues from 520 to 633) preferentially recognizes asymmetrically dimethylated peptides over symmetrically dimethylated peptides and monomethylated peptides, consistent with two recent reports [21], [23]. Therefore, TDRD3 has a different binding selectivity than SND1, which we have previously established that the extended Tudor domain of SND1 preferentially binds symmetrically dimethylated arginine PIWIL1 peptides [20]. Similar to SND1, the binding selectivity of TDRD3 among these three different arginine methylation marks is about 2 to 4-fold (Table 1) [20].

**Table 1:** Binding affinities of TDRD3 (aa 520–633), SMN (aa 80–170), and SPF30 (aa 65–150) to different methyl-arginine peptides in comparison with SND1 (aa 650–910).

Peptide Name	Amino Acid Sequence	Kd (µM)			
		TDRD3 (aa 520–633)	SMN (aa 80–170)	SPF30 (aa 65–150)	SND1 (aa 650–910)
PIWIL1-R4un	TGRARARARGRARGQE	>500	>300	>500	94±6*
PIWIL1-R4me1	TGRmeARARARGRARGQE	>500	>150	>500	19±1*
PIWIL1-R4me2a	TGRme2aARARARGRARGQE	>150	97±3	>400	42±2*
PIWIL1-R4me2s	TGRme2sARARARGRARGQE	>300	34±2	>200	10±1*
SmD3RSDMA	GGRGRme2sGRG	>400	94±4	NB	56±8
PGM(SmB165SD)	YPPGRme2sGGPPP	NB	>300	NB	>500
PGM(SmB214SD)	PPGMRme2sPPPPG	NB	>400	NB	NB
PGM(SmB221SD)	PPGMRme2sGPPP	NB	>500	NB	NB
H3R2me1	ARmeTKQTARKSY	NB	>300	NB	>300
H3R2me2a	ARme2aTKQTARKSY	>500	>200	NB	>300
H3R2me2s	ARme2sTKQTARKSY	>500	>150	NB	99±6*
H3R17me2a	TGGKAPRme2aKQLATKA	>1000(ITC)			
H3R17me2s	TGGKAPRme2sKQLATKA	NB(ITC)			
H4R3me2a	SGRme2aGKGGK	NB(ITC)			
Histone H3K4me1/2/3	ARTK (me)1/2/3 QTARKST	NB	NB	NB	NB*
Histone H3K9me1/2/3	ARTKQTARK(me)1/2/3STGGKA	NB	NB	NB	NB*

NB: No detectable binding.

*: data from Liu K et al, PNAS, 2010 [20].

For SND1, we found that the canonical Tudor domain of SND1 is not sufficient for binding its ligands. Its N-terminal and C-terminal extensions, which fold together to form another Tudor-like domain, are required for binding the methyl-arginine PIWIL peptides [20]. Thus, we asked whether the Tudor domain of TDRD3 is capable of binding its ligands by itself. To this end, we used purified protein from our crystallization construct (residues from 553 to 611), which only covers the canonical Tudor domain, to test if the binding affinity is abolished, and found that the crystallization construct has almost the same binding affinity as the longer construct (residues from 520 to 633). Hence, in regards to TDRD3, its Tudor domain is sufficient for ligand binding.

**Figure 2:**
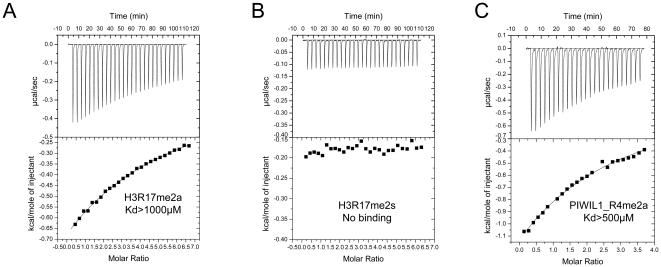
Preferential binding of TDRD3 to asymmetrical dimethylated H3R17 peptide over symmetrical dimethylated H3R17 peptide. The ITC measurements were done in 20 mM Tris pH 8.0 and 200 mM NaCl using the same TDRD3 construct as used in Table 1. The measurements were taken at 25°C. Binding isotherms were plotted and analyzed using Origin Software (MicroCal Inc.). The ITC measurements were fit to a one-site binding model. (A) Histone H3R17me2a peptide (TGGKAPRme2aKQLATKA). (B) Histone H3R17me2s peptide (TGGKAPRme2sKQLATKA). (C) PIWIL1_R4me2a peptide (TGRme2aARARA).

Recently, it has been suggested that the Tudor domain of TDRD3 preferentially recognizes H3R17me2a and H4R3me2a peptides and promotes transcription by binding these methylarginine marks [21], [23]. Our ITC (Isothermal Titration Calorimetry) binding results show that TDRD3 preferentially recognizes H3R17me2a over H3R17me2s (Fig. 2), which is consistent with our fluorescence polarization results for other methyl-arginine peptides (Table 1). Very interestingly, the R17 residue does not reside in a GAR motif, which is also the case in the methylated Pol II CTD [23]. Therefore, the Tudor domain of TDRD3 does not only bind GAR motif but also other motifs. In comparison, we also measured the binding affinity of the PIWIL1_R4me2a peptide with TDRD3 at the same conditions by ITC, which binds modestly tighter than the H3R17me2a peptide (Fig. 2). On the other hand, TDRD3 does not bind the methyl-arginine at the PGM motifs (Table 1). Taken together, the Tudor domain of TDRD3 preferentially binds asymmetrically dimethylated peptides with a preference for GAR motifs.

### Crystal structures of TDRD3 in complex with two methyl-arginine mimics

**Figure 3:**
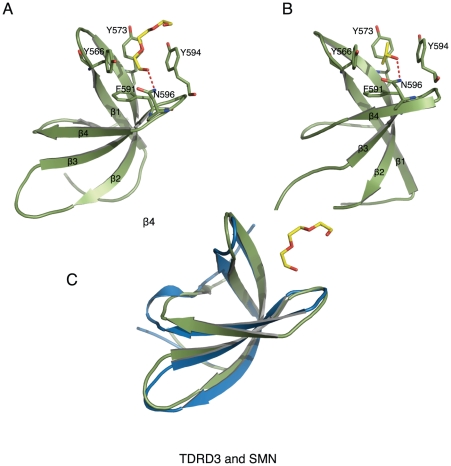
Crystal structures of TDRD3 with methyl-arginine mimics. (A) TDRD3 in complex with tetraethylene glycol (PG4). (B) TDRD3 in complex with isopropanol. The aromatic cage residues and small molecules are displayed in a stick model. (C) Superposition of the crystal structures of TDRD3 and SMN (PDB: 1MHN). The tetraethylene glycol molecule is shown in a stick model. SMN is colored in blue and TDRD3 is colored in green.

In order to better understand the molecular mechanism of methyl-arginine binding by the Tudor domain of TDRD3, we tried cocrystallization of the TDRD3 Tudor domain with different methyl-arginine peptides. Although we could not obtain cocrystals of TDRD3 with any of these peptides, we found a tetraethylene glycol (PG4) or isopropanol (2-propanol) molecule in our crystal structures. These compounds are from our crystallization solutions. Interestingly, these compounds bind to TDRD3 and occupy the aromatic cage of TDRD3 (Fig. 3A and 3B).

**Figure 4:**
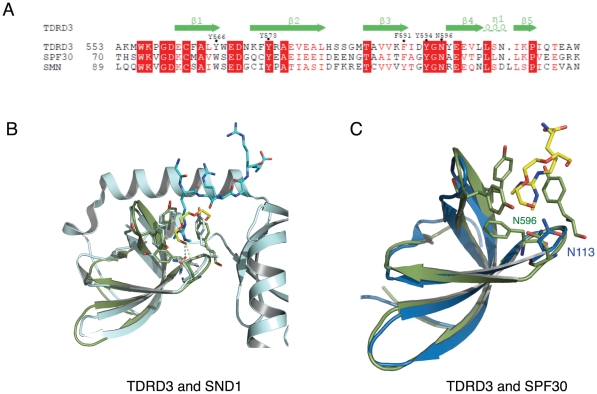
Structural comparison of TDRD3 to SND1 and SPF30. (A) Structure-based sequence alignment of the Tudor domain of TDRD3, SPF30 and SMN. The aromatic cage residues are denoted by black dots. Identical residues are colored in white on red background, and similar residues are colored in red. (B) Superposition of the crystal structures of TDRD3 and SND1 (PDB: 3OMC). SND1 is colored in cyan and its bound ligand PIWIL1_R4me2s peptide is shown in a stick model. (C) Superposition of the crystal structures of TDRD3 and SPF30 (PDB: 4A4F). The tetraethylene glycol molecule and methyl-arginine are shown in a stick model. SPF30 is colored in blue and TDRD3 is colored in green.

The overall structure of the Tudor domain of TDRD3 is very similar to that of the SMN Tudor domain with an RMSD of 1.1 Å for all aligned Cα atoms (Fig. 3C). The TDRD3 and SMN Tudor domains have a sequence identity of 37% (Fig. 4A). Consistent with the SMN structure, the Tudor domain of TDRD3 exhibits a five-stranded β-barrel fold (Fig. 3A). The tetraethylene glycol or isopropanol molecule is bound in an aromatic rectangle cuboid cage formed by the aromatic residues Y566, Y573, F591 and Y594, reminiscent of the methylarginine binding by the SND1 Tudor domain [20] or *Drosophila* Tudor [19] (Fig. 3). The tetraethylene glycol molecule exhibits a linear conformation, parallel to the aromatic rings of residues Y566, Y573 and Y594 and perpendicular to residue F591 (Fig. 3A). Likewise, the isopropanol molecule is flanked by the aromatic rings of residues Y566 and Y594 with the hydrogen from the CH group pointing to the aromatic ring of residue Y566 (Fig. 3B). Furthermore, by superimposing the Tudor domain structures of TDRD3 with those of the SND1-PIWIL1 peptide structures and the recently released SMN/SPF30-methyl-arginine residue structures [24], we found that the small molecules (PG4 or isopropanol) reside in a similar position to the side chain of the methyl-arginine (Fig. 4B and 4C).

In the SND1 and PIWIL1 peptide complex structures, besides the aromatic cage, the methyl-arginine also forms a hydrogen bond with residue N768 through the NH1/2 group, and disruption of this hydrogen bond severely diminishes the binding [20]. Interestingly, in both the tetraethylene glycol and isopropanol complex structures, the conserved asparagine N596 also forms a hydrogen bond with the tetraethylene glycol or isopropanol molecule, respectively. Thus, both the tetraethylene glycol and the isopropanol molecule are bound by the aromatic cage and residue N596 of TDRD3 through hydrophobic and hydrogen bonding interactions. Nevertheless, during the submission of the manuscript, NMR structures of SMN and SPF30 in complex with methyl-arginine residues were released [24]. These newly released structures show that SMN and SPF30 do not form hydrogen bonds with their ligands (Fig. 4C). In the SMN and SPF30 structures, the methyl groups are attached to arginine in a different configuration, which presents hydrogen bond formation between the symmetrically dimethylated arginine and the conserved asparagine (N132 in SMN and N113 in SPF30). In addition, the dimethylated arginine pushes the asparagine away, which points to solvent in the SMN and SPF30 structures (Fig. 4C).

Previously, we have demonstrated that SND1, 53BP1, and L3MBTL1/2 all have similar aromatic cages [17], [19], [20], [25], [26], [27]. 53BP1 and L3MBTL1 selectively bind low methylation states of lysine in histone tails, but SND1 selectively recognized arginine methylated peptides. By comparing the aromatic cage dimensions, it was found that the distance between the F740 and Y766 in SND1 is 1.2 Å narrower than that between the Y1502 and Y1523 in 53BP1 [20]. The narrower cage size in the extended Tudor domain of SND1 favors the planar methyl-guanidinium group. By comparing the Tudor domain structure of TDRD3 and SND1, it was found that the aromatic cage has a very similar size to that of SND1 (Fig. 4B), which explains why TDRD3 selectively binds methyl-arginine proteins, but not methyl-lysine proteins (Table 1).

### SMN preferentially recognizes symmetrically dimethylated peptides

SMN protein is a core component of the SMN complex, which plays an essential role in spliceosomal snRNP assembly in the cytoplasm and is required for pre-mRNA splicing in the nucleus [28]. Recessive mutations in the SMN1 gene cause all four types of spinal muscular atrophy disorders (SMA1–4). Dreyfuss's laboratory showed that SMN preferentially binds to the dimethylated GAR motifs of SmD1 and SmD3, and methylation also promotes its interaction with other SMN-interacting proteins [4]. Peptide competition assay by Brahms *et al* implicated that SMN preferentially binds symmetrically dimethylated Sm proteins D1/D3, B/B′ and the Sm-like protein LSm [29]. Symmetrical dimethylation of the Sm proteins is carried out by PRMT5 and PRMT7 [30], [31]. Whitehead *et al* argued that arginine dimethylation is not required for SMN recognition of proteins bearing GAR motifs, although they agreed that GAR motif is essential in SMN binding [32]. In another study, it was also shown that the Tudor domain of SMN interacts with the EWS protein (Ewing's sarcoma protein) via its GAR motifs, but symmetrical dimethylation reduces this interaction [33]. In addition to the GAR motifs, SMN is also able to bind the PGM motifs of CA150, SmB, and other splicing factors in a CARM1-dependent fashion. CARM1 carries out arginine monomethylation and asymmetric dimethylation [6]. In order to reconcile the differences among these reports, we systematically characterized the binding property of SMN using our fluorescein-labeled peptide library by means of fluorescence polarization binding assays.

Our binding results show that SMN preferentially recognizes symmetrically dimethylated arginine peptides (Table 1). It binds the symmetrical dimethylated peptide of the PIWIL1 protein (PIWIL1_R4me2s) with a Kd of 34 µM. The binding affinity was about 3 times weaker for the asymmetrical dimethylated PIWIL1 peptide (PIWIL1_R4me2a, Kd = 97 µM), over 4 times weaker for the monomethylated PIWIL1 peptide (PIWIL1_R4me1, Kd>150 µM). Interestingly, SMN also binds the unmethylated PIWIL1 peptide (Table 1), consistent with some reports suggesting that arginine methylation is not crucial for binding GAR motif containing proteins [32], [33]. A similar trend holds for the different modifications of histone H3R2 peptides, which does not have a GAR motif. In agreement with the fact that SMN binds PGM motif containing proteins in a methylation-dependent manner, SMN is also able to bind the sDMA PGM motifs from the splicing protein SmB, *albeit* with lower affinity in comparison to the GAR motif containing SmD1/3 and PIWIL1 peptides. Like TDRD3, the Tudor domain of SMN is sufficient for binding. The SMN construct covering only the Tudor domain (residues 82–147) binds to the PIWIL1_R4me2s peptide with a Kd of 46 µM. Taken together, SMN is a very promiscuous effector molecule, which preferentially binds symmetrical dimethylated arginine via its Tudor domain.

**Figure 5:**
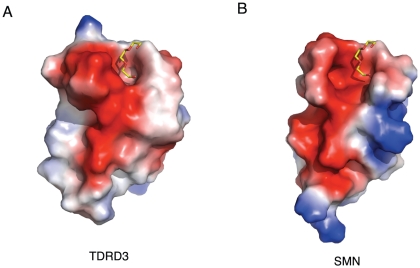
Surface representation of TDRD3 and SMN. (A) TDRD3 (B) SMN (PDB: 1MHN). The ligand is superimposed from the TDRD3-PG4 structure.

In this study, we report the high-resolution crystal structures of the Tudor domain of TDRD3, and the high-resolution structure of SMN has been reported previously [22], [34]. By comparing the ligand binding grooves of these two proteins (Fig. 5), we found that SMN displays a much wider binding groove near the aromatic cage, which could potentially explain why SMN is a very promiscuous effector molecule binding different motifs, especially PGM motifs. Proline acts as a secondary structural element disruptor, and is often found in turns. In order to accommodate the proline-rich PGM motifs, it is conceivable that a larger binding groove, such as that identified in the SMN Tudor domain, is essential. That explains why SMN is a very promiscuous effector molecule.

SPF30, a homolog of SMN, also contains a Tudor domain, which has a 45% sequence identity with the SMN Tudor domain (Fig. 4A). Our binding results show that SPF30 only binds the GAR motif containing PIWIL1 peptides with a lower affinity in comparison to TDRD3 and SMN (Table 1). Therefore, although TDRD3, SMN and SPF30 all contain a conserved Tudor domain, they exhibit different binding properties.

In summary, in this study, we systematically characterized the binding specificity and affinity of the Tudor domains of TDRD3, SMN, and SPF30 quantitatively, which show that TDRD3 preferentially recognizes asymmetrical dimethylated arginine mark, and SMN is a very promiscuous effector molecule, which recognize different arginine containing sequence motifs and preferentially binds symmetrical dimethylated arginine. SPF30 is the weakest methyl-arginine binder, which only binds the GAR motif sequences. These Tudor domains have been reported to exhibit weak binding affinity (mM scale) to SmD3 methyl-arginine peptides by NMR titration [22], which is significantly lower compared to other methyl-lysine/arginine Tudor binders, such as JMJD2A [16], 53BP1 [17], SGF29 [35] and SND1 [20], but comparable to FXR1/2 [36] and PWWP proteins [37] By peptide screening, we identified some higher affinity ligands for the TDRD3 and SMN Tudors, which means that ligands of stronger binding affinities with these Tudor domains potentially exist, which warrants further investigation in the future. In addition, we report high resolution crystal structures of the Tudor domain of TDRD3 with two methyl-arginine mimics, which provides the first glimpse of methyl-arginine binding by these Tudor domains.

## Materials and Methods

### Protein preparation and crystallization

Different length of TDRD3, SMN and SPF30 fragments covering the Tudor domain were amplified from human cDNA and sub-cloned into pET28-MHL vector. The recombinant proteins were overexpressed at 14°C in Escherichia coli BL21 (DE3) overnight as N-terminal His-tagged fusion proteins. Freezed Cell pellets were re-suspended in 200 ml lysis buffer (1×PBS, pH 7.2–7.4, 250 mM NaCl, 5% glycerol) for every 40 g cell pellet. Adding 20 unit Benzoate Nuclease, 0.2 mM PMSF, 0.2% CHAPS, 20 mM β-mercaptoethanol and were purified by affinity chromatography on Ni-NTA. TDRD3 protein used for crystallization was cleaved using TEV by incubating at 4°C for overnight. All of the eluted proteins were collected and purified further by size exclusion chromatography (HiLoad 16/60 Superdex 75, GE) in a buffer of 20 mM Tris pH 7.0, 200 mM, 1 mM DTT, respectively. Protein was concentrated to 10 mg/ml and crystallized by sitting-drop vapor diffusion. The PG4 cocrystal was grown at 0.1 M Tris-HCl pH 8.5, 2.0 M Ammonium sulfate and PEG400 additive at 18°C. The isopropanol crystal was grown in a condition with 10% 2-propanol, 100 mM Phosphate-Citrate pH 4.0 and 0.2 M Li_2_SO_4_.

### Peptide binding Assays

All the regular and fluorescent peptides used this study were synthesized by Tufts University Core Services (Boston, USA). The fluorescence polarization assay was carried out as described before [38]. The buffer used in the fluorescence polarization assay is 20 mM Tris pH 7.5, 50 mM NaCl, 1 mM DTT and 0.01% Triton X-100. An excitation wavelength of 485 nm and an emission wavelength of 528 nm are used. The data are measured at 25°C and corrected for background by subtracting the free-labeled peptide background. The data were collected by the Synergy 2 (BioTec, USA) fluorescence polarization program and were fit to one-site binding model using Origin 7 (MicroCal, Inc.). The Kd values are the average of three independent measurements.

The protocol for ITC (Isothermal Titration Calorimetry) was carried out as described before [39]. The ITC buffer used in this study is 20 mM Tris pH 8.0 and 200 mM NaCl. The measurements were taken at 25°C. Binding isotherms were plotted and analyzed using Origin Software (MicroCal Inc.). The ITC measurements were fit to a one-site binding model.

### Data collection, structure determination and refinement

Diffraction data for the TDRD3-PG4 crystal were collected at 100 °K using CuKα radiation generated on a Rigaku FR-E SuperBright rotating anode system equipped with VariMax HF optics and a Saturn A200 CCD detector. Data were integrated and scaled using the HKL2000 software package [40]. The structure of the Tudor domain of human Tudor domain-containing protein 3 was solved using the single-wavelength anomalous dispersion (SAD) method [41] utilizing the anomalous signal from one sulfur atom corresponding to a highly ordered Cys residue present in the crystal. The position of the sulfur anomalous scatterer was determined using SHELXD [42], followed by heavy-atom refinement and maximum-likelihood-based phasing as implemented in the autoSHARP program suite [43]. Phase improvement by density modification generated an interpretable experimental electron density map, which allowed an initial model of the polypeptide chain to be traced using ARP/wARP [44]. Following several alternate cycles of manual rebuilding using COOT [45] and restrained refinement against a maximum likelihood target, the improved model revealed clear electron densities allowing placement of water molecules. All refinement steps were performed using REFMAC [46] in the CCP4 proegram suite suite. During the final cycles of model building, TLS parameterization [47] was included in the refinement of the final model which comprised protein and solvent molecules. The diffraction data for the TDRD3-isopropanol crystal was collected on a Rigaku 007 generator and a R-AXIS detector. The structure was determined by molecular replacement using Molrep [48] and refined in a similar protocol to the TDRD3-PG4 structure. The data collection and refinement statistics are summarized in Table 2.

**Table 2:** Data collection and refinement statistics.

	TDRD3-PG4	TDRD3-Isopropanol
**Data collection**		
Space group	*P*6_5_	*P*6_5_
Cell dimensions		
*a*, *b*, *c* (Å)	41.7, 41.7, 59.9	41.9, 41.9, 61.5
α, β, γ (°)	90, 90, 120	90, 90, 120
Wavelength (Å)	1.5418	1.5418
Resolution (Å)	50.0–1.80 (1.84–1.80)	36.0–1.78 (1.84–1.78)
*R*_merge_ # (%)	3.1 (9.9)	3.1 (6.1)
*<I>/<σ(I)>* *	104.4 (14.4)	62.5 (39.2)
Completeness (%)	98.7 (85.8)	98.4 (100.0)
Redundancy	19.5 (8.5)	9.7 (9.5)
**Refinement**		
Resolution (Å)	19.70–1.80	36.0–1.78
No. reflections	5,444	5,564
*R*_work_/*R*_free_	17.7/20.9	19.1/20.9
No. atoms		
Protein	451	443
Ligand	10	8
Water	36	67
Average B-factors (Å^2^)		
Protein	20.7	13.0
Ligand	36.6	19.4
Water	32.2	31.5
R.m.s. deviations§		
Bond lengths (Å)	0.010	0.008
Bond angles (°)	1.16	1.06

*: Values in parentheses correspond to the highest resolution shells.

# *R_merge_ = *Σ*_hkl_*Σ*_j_*∣*I*(*hkl;j*)−<*I*(*hkl*)>∣/(Σ*_hkl_*Σ*_j_*<*I*(*hkl*)>), where *I*(*hkl;j*) is the *j*th measurement of the intensity of the unique reflection (*hkl*), and *I*(*hkl*) is the mean overall symmetry related measurements.

§ R.m.s. deviations: root mean squared deviation.

## Supporting Information

Figure S1 **Binding affinities of TDRD3 to PIWIL1 peptides at 200 mM NaCl.** The buffer used in the fluorescence polarization assay is 20 mM Tris pH 7.5, 200 mM NaCl, 1 mM DTT and 0.01% Triton X-100. The data are measured at 25°C and corrected for background by subtracting the free-labeled peptide background. The Kd values are the average of three independent measurements.
(EPS)
